# Reflecting on 12 years of training medical students in rural longitudinal integrated clerkships

**DOI:** 10.4102/phcfm.v16i1.4390

**Published:** 2024-04-22

**Authors:** Francois Coetzee, Ian Couper

**Affiliations:** 1 Ukwanda Centre for Rural Health, Faculty of Medicine and Health Sciences, Stellenbosch University, Stellenbosch, South Africa

**Keywords:** longitudinal integrated clerkships, medical training, primary health care environments, final year students, rural health care

## Abstract

Longitudinal integrated clerkships (LICs) are effective in promoting careers in rural primary health care environments. This model of training medical professionals involves longer clinical placements of medical students and a different approach to learning which better prepares them for primary health care practice. Stellenbosch University created a LIC in 2011 for this purpose and has trained almost 100 doctors in their yearlong LIC since then. The past 12 years have brought about a lot of learning as this model of training was implemented, developed, and refined to suit the needs of students and the clinical environments.

**Contribution:**

Countries across the globe face challenges in recruiting and retaining doctors in rural primary health care environments. Longitudinal integrated clerkships have several educational benefits in addition to increase recruitment and retention of rural doctors, and 12 years of experience have led to a greater understanding regarding implementation and outcomes of an LIC in the South African context.

## Why we started

The Faculty of Medicine and Health Sciences (FMHS) at Stellenbosch University pioneered a year-long longitudinal integrated clerkship (LIC) for medical students in South Africa when it opened a Rural Clinical School (RCS) in 2011.^1^ There is sufficient evidence that this model of training provides significant educational benefits,^2,3,4^ together with being an effective intervention to recruit and retain clinicians to work in rural environments.^5,6^ A large investment of time and resources was made to develop this LIC, as this model of training aligned well with the FMHS’s vision of training fit-for-purpose clinicians who would contribute towards equity in health care and to increase access to high quality health care for rural communities.^7^

## Basis for the model

Longitudinal integrated clerkships are very different from the traditional clinical block rotations of a few weeks in a hospital, as it places students in clinical environments for 6 months or more. This longitudinal placement allows students to be integrated into clinical teams, build learning relationships with peers, preceptors and patients, gain confidence in the management of undifferentiated patients, and get a better understanding of the health care system.^8^ Longitudinal integrated clerkships can take on various formats, occur in a range of contexts, and may be undertaken during any of the years of a medical curriculum, though commonly in the pre-final or final year.^9^ A LIC for medical students fulfils the following criteria:

… students participate in the comprehensive care of patients over time, have continuing learning relationships with these patients’ clinicians and through these experiences, medical students meet the majority of the academic year’s core clinical competencies across multiple disciplines simultaneously.^9^

## What we do

Our LIC, which we call the ‘longitudinal integrated model’ (LIM), takes place in the final year of a 6-year MBChB curriculum. The LIM allows a small group of students to be trained in rural primary health care environments. At the beginning of the fifth year, students apply for a final year placement at a LIM site and, after a selection process, they are allocated a placement at one of the available sites while attempting to incorporate site and clinical partner preferences of the students.

At the sites, students are required to work alongside health care professionals in clinical teams, using integrated, patient-based learning. Students regulate their own learning and decide with their site supervisors how they will spend their time in clinical environments. They are encouraged to balance working in focused environments (theatre, the paediatric ward and the labour ward) with working in generalist environments (the emergency centre, the out-patients department and primary health care clinics). Although we do not specify the exact working arrangements hour by hour, students are expected to work a full working day comparable to the doctors at the facility with the exception of fewer and shorter after-hour calls. All but one of our sites are district hospitals run by generalist medical officers and family physicians. The exception is an under-resourced regional hospital with specialist departments, where we have a modified LIM, in which time spent in focused and generalist environments is pre-determined, while longitudinal exposure to primary health care and integrated learning is maintained.

Students are supported academically by means of weekly input from clinical domain specialists who engage with the LIM students in online discussions about patients they have been involved with. Faculty provide additional support during site visits, by facilitating reflections on learning experiences and by guiding students regarding the optimal use of the available learning opportunities.

Longitudinal integrated model students have to write up 58 patients during the year, of which there is a required number specified for each of the following clinical domains: primary health care, psychiatry, orthopaedic surgery, general surgery, internal medicine, obstetrics and gynaecology, and paediatrics. Each patient write-up or ‘patient study’ is a self-directed learning project. Students select suitable patients from those they encounter that will address their learning needs in relation to the outcomes as specified in the study guides. For primary health care, the students have to acquire a portfolio of patients with chronic diseases or persistent clinical problems that they follow up over the entire year. For each of these patients, a student must have at least three encounters, one of them being a contextual or home visit.

## Where we do it

**FIGURE 1:**
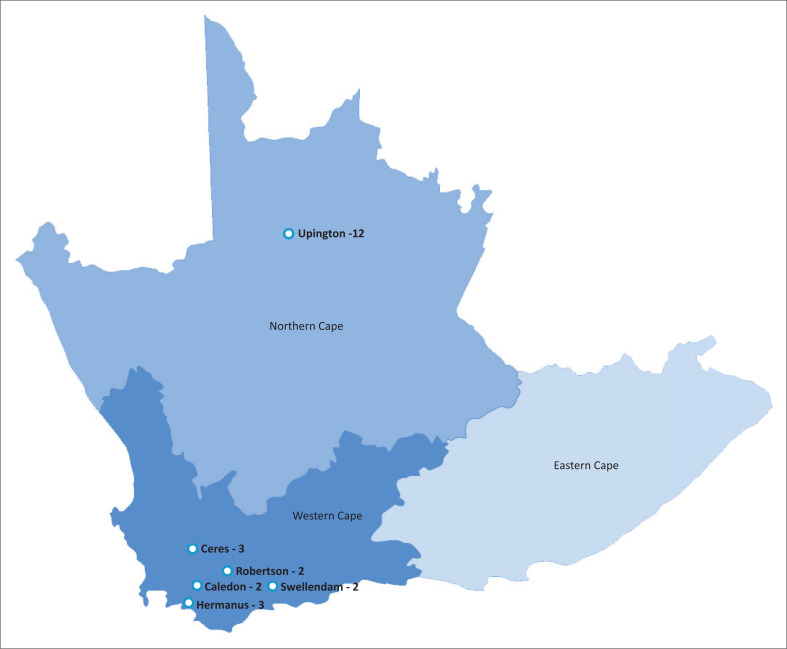
A map of the longitudinal integrated model sites and the number of students that are placed at them.

Current LIM sites include district hospitals in the Western Cape (Ceres, Robertson, Hermanus, Swellendam and Caledon Hospitals) and a regional hospital in the Northern Cape (Dr Harry Surtie Hospital in Upington). The sites and student numbers have expanded gradually from placing two students at Ceres in 2011, to placing students at almost all the above-mentioned sites every year. (See Figure 1 for a map indicating the sites and the current maximum number of students placed at each site.)

## What we have learnt

The LIM is in the final year and therefore it offers students an opportunity to integrate what they have learnt in the previous years into practice. Students are also afforded opportunities to develop their professional identities and procedural skills at an appropriate time in their professional development.^8,10^ Although there are numerous learning opportunities and supportive relationships available in the LIM, for the first 5 years of their course, students have been trained in a tertiary hospital environment that is vastly different from primary health care environments, so there is often a significant adjustment period for students, during which they experience high levels of discomfort.^11,12,13^ Because of this challenge, the selection process in earlier years allowed only students with strong academic records to participate in this model of training. However, over time, we have learnt that the LIM also allows for students with challenging academic records to flourish and surpass expectations regarding their capabilities and professional development. From our experience it appears that a positive disposition towards primary health care, patient-based learning and self-regulated learning are better predictors of students’ success than their academic history. At the very least, we know that the students are not disadvantaged academically.^14^

Longitudinal placement in a clinical setting allows LIM students to form meaningful learning relationships and raises the ceiling of what they can learn significantly higher compared to students in traditional block rotations.^8^ We have been pleasantly surprised on several occasions by what students can achieve during their training. Some students excel in developing their procedural skills beyond what is expected of a final year student, while others become very involved in helping to sort out complex biopsychosocial patient problems. Some develop a sense of responsibility that causes them to go beyond the required working hours, necessitating that faculty remind them that they cannot always remain after hours to help out the team. Similarly, some students go beyond what we expect regarding inter-professional collaboration and the number of contextual patient visits performed. We suspect that, as described Walters et al.,^15^ the possibility of developing better personal connections with patients might explain why students trained within an LIC are more likely to incorporate primary health care concepts such as patient-centred practice, holistic care, and interprofessional collaboration into their daily practice compared to their peers trained in traditional block rotations.^15^

Regarding faculty development for preceptors at the LIM sites, we have learnt that it must be approached with a high degree of flexibility. Members of the healthcare team, including all the health care professionals at the facility, are not required to ‘teach’ students as would typically be the practice in an academic ward round, but they are encouraged to facilitate the students’ learning by asking questions to focus their attention on the pertinent issues. They are also required to provide input in the workup and care of patients which students are responsible for.

It is essential to provide adequate support to both students and the local clinician-preceptors. Students receive logistics and accommodation support from the university’s logistics unit, SUNLOC and academic support from the RCS faculty, specialist consultants at the regional hospital and the academic units at the central teaching hospital. Most of the academic support is provided via online meetings, where approaches to common clinical problems linked to student–patient encounters are discussed. Students also have access to the online resources of the academic units. Faculty development is provided annually at the beginning of the year to ensure that newly appointed clinicians are oriented to the LIM, relationships with local clinicians are maintained, and any programme changes are communicated to the sites. This training is followed up by a number of site visits during the year to support student learning. As few clinicians are available for off-site activities, we have also used site visits as an opportunity for faculty development of clinicians unfamiliar with the programme. Bringing training into district hospitals evokes a new sense of identity for clinicians as educators and their own professional learning is enhanced as a result of supervising students.^16^

## Where are we going?

The RCS and the FMHS have learned valuable lessons regarding clinical training in rural primary health care environments as outlined earlier in the text. Our experiences have influenced curriculum design for medical and health sciences students at Stellenbosch University. Examples include the creation of a longitudinal primary health care experience in the first 3 years of the renewed MBChB curriculum and a LIM within the physiotherapy curriculum. Continued efforts are made to expand the LIM sites and the number of students placed in LIM; two additional LIM sites are planned for 2025.

In the final year of the renewed curriculum, known as the Distributed Clinical Apprenticeship, the entire class will be trained in clinical environments outside of the central teaching hospital. A larger portion of the final year class will have the option of being trained in rural environments in a model similar to the LIM, while all of the final year students will work in primary health care environments for at least 6 months.

In the short-term, the assessment of students in LIM will change in 2024, with the use of integrated assessments that align better with the learning experience of students, rather than the previous domain-based, siloed assessments that are mismatched with the LIM approach.^17^

Longitudinal clinical exposure to primary health care environments has significant educational and workforce benefits and will therefore continue to be an important educational strategy locally and globally.

We are passionate about LICs and are eager to support those who want to create similar models of training; those interested can contact the corresponding author (FC) and visit our resource page at https://rb.gy/pk0cuk.

We would like to acknowledge and are very grateful to all the professional administration staff and clinicians that have been involved in making the Longitudinal Integrated Model a great success during the past 12 years.
